# Withdrawal: Pigment Epithelium-derived Factor Inhibits Angiogenesis via Regulated Intracellular Proteolysis of Vascular Endothelial Growth Factor Receptor 1

**DOI:** 10.1016/j.jbc.2022.102346

**Published:** 2022-08-12

**Authors:** Jun Cai, Wen G. Jiang, Maria B. Grant, Mike Boulton

This article has been withdrawn at the request of the authors except Jun Cai and Wen G. Jiang who could not be reached by the journal. The journal analysis and authors conclude that the α-tubulin panel in Figure 2a has been flipped horizontally and reused as VEGFR-1 panel in Figure 2d. Due to the lack of original research records, the authors wish to withdraw this article.

